# Draft Genome Sequence of *Sphingobium lactosutens* Strain DS20^T^, Isolated from a Hexachlorocyclohexane Dumpsite

**DOI:** 10.1128/genomeA.00753-13

**Published:** 2013-09-19

**Authors:** Roshan Kumar, Vatsala Dwivedi, Vivek Negi, J. P. Khurana, Rup Lal

**Affiliations:** Molecular Biology Laboratory, Department of Zoology, University of Delhi, Delhi, Indiaa; Interdisciplinary Centre for Plant Genomics & Department of Plant Molecular Biology, University of Delhi South Campus, New Delhi, Indiab

## Abstract

*Sphingobium lactosutens* DS20^T^ has been isolated from the hexachlorocyclohexane (HCH) dumpsite in Lucknow, India, but does not degrade any of the HCH isomers. Here, we present the ~5.36-Mb draft genome sequence of strain DS20^T^, which consists of 110 contigs and 5,288 coding sequences, with a G+C content of 63.1%.

## GENOME ANNOUNCEMENT

*Sphingobium lactosutens* strain DS20^T^ was isolated from the hexachlorocyclohexane (HCH)-contaminated dumpsite located at Lucknow, Uttar Pradesh, India (1). In order to understand the evolution and acquisition of *lin* genes responsible for the degradation of hexachlorocyclohexane isomers, we have sequenced the genomes of several sphingomonads isolated from this HCH dumpsite (2, 3, 4, 5). All of these sphingomonads which possess *lin* genes were able to degrade HCH isomers to various degrees. However, despite the fact that strain DS20^T^ tolerated HCH levels as high as 450 mg/g of soil (6), it did not degrade any of the HCH isomers.

The genomic DNA was sequenced by the Beijing Genome Institute (BGI) (Hong Kong) by using a combined approach with the 454 GS FLX Titanium and Illumina HighSeq 2000 systems with a paired-end (2 kb) library. The total raw data generated consisted of 761 Mbp, which finally led to 597 Mbp of clean data. The reads generated were assembled using ABySS 1.3.3 (7). The assembly was further validated based on the paired-end information by using the Burrows Wheeler aligner (8). Automatic gene annotation was performed using RAST version 4.0 (9) and the NCBI Prokaryotic Genomes Annotation Pipeline (PGAP) version 2.0 (http://www.ncbi.nlm.nih.gov/genomes/static/Pipeline.html).

A total of 110 contigs (*N*_50_, 29.6) covering a total of 5,360,246 bp were generated at a k-mer of 53. The largest contig assembled was approximately 516 kb. The draft genome has a GC content of 63.1%. The genome consists of 5,288 protein-coding genes. Fifteen rRNA genes and 59 tRNA genes were identified. The presence of genes encoding plasmid initiator proteins and *par* genes, as well as genes involved in conjugative transfer in the draft genome, indicates the occurrence of extrachromosomal elements. One clustered regularly interspaced short palindromic repeat (CRISPR) element was identified in contig number 109 by using CRISPR Finder (10). The size of the CRISPR element is 360 bp, with a direct repeat (DR) length of 32 bp along with 5 spacers.

Genes responsible for degradation of phenol/toluene, chlorophenol, anthranilate, and homogentisate were also identified. In addition, more than 40 mobile genetic elements, mostly contributed by IS*6100*, ISSsp*5*, and ISSpma*1* elements, were identified (11). In-depth analysis of the draft genome revealed the absence of *lin* genes of the upper HCH degradative pathway (12) in DS20^T^. Interestingly, some of the *lin* genes of the lower HCH degradative pathway, *linKLMN*, which code for the ABC transporter system, are present. This reflects that DS20^T^, like other sphingomonads, has a minimal transporter system and in due course of time may acquire other *lin* genes to develop a complete HCH degradation pathway.

The genome sequence of strain DS20^T^, which does not yet degrade any of the HCH isomers and has partially acquired the *lin* genes of the lower HCH degradation pathway, thus becomes a valuable source for performing comparative genomic studies by using already available draft genomes of several HCH-degrading sphingomonads (2, 3, 4, 5) and metagenomic data (13, 14).

### Nucleotide sequence accession numbers.

The draft genome sequence of DS20^T^ is available in GenBank under the accession number ATDP00000000. The version described in this paper is the first version, ATDP01000000.
